# Glycan Epitope and Integrin Expression Dynamics Characterize Neural Crest Epithelial-to-Mesenchymal Transition (EMT) in Human Pluripotent Stem Cell Differentiation

**DOI:** 10.1007/s12015-022-10393-1

**Published:** 2022-06-21

**Authors:** Ria Thomas, Vishal Menon, Rakesh Mani, Jan Pruszak

**Affiliations:** 1 Emmy Noether-Group for Stem Cell Biology, Department of Molecular Embryology, Institute of Anatomy and Cell Biology, Faculty of Medicine, University of Freiburg, Freiburg, Germany; 2 Spemann Graduate School of Biology and Medicine and Faculty of Biology, University of Freiburg, Freiburg, Germany; 3 Neuroregeneration Research Institute, McLean Hospital/ Harvard Medical School, Belmont, MB USA; 4 Wellcome Trust/ Cancer Research UK Gurdon Institute, University of Cambridge, Cambridge, UK; 5 Institute of Anatomy and Cell Biology, Salzburg, Paracelsus Medical University (PMU), Salzburg, Austria; 6 Center of Anatomy and Cell Biology, Salzburg and Nuremberg, Paracelsus Medical University (PMU), Salzburg, Austria

**Keywords:** Pluripotent stem cells, Neural development, Neural crest, Epithelial-to-mesenchymal transition (EMT), Surface molecules, Cluster-of-differentiation (CD) antigens, Biomarkers, Glycoproteins, Integrins

## Abstract

The neural crest gives rise to progeny as diverse as peripheral neurons, myelinating cells, cranial muscle, bone and cartilage tissues, and melanocytes. Neural crest derivation encompasses complex morphological change, including epithelial-to-mesenchymal transition (EMT) and migration to the eventual target locations throughout the body. Neural crest cultures derived from stem cells provide an attractive source for developmental studies in human model systems, of immediate biomedical relevance for neurocristopathies, neural cancer biology and regenerative medicine, if only appropriate markers for lineage and cell type definition and quality control criteria were available. Implementing a defined, scalable protocol to generate neural crest cells from embryonic stem cells, we identify stage-defining cluster-of-differentiation (CD) surface markers during human neural crest development *in vitro*. Acquisition of increasingly mesenchymal phenotype was characterized by absence of neuroepithelial stemness markers (CD15, CD133, CD49f) and by decrease of CD57 and CD24. Increased per-cell-expression of CD29, CD44 and CD73 correlated with established EMT markers as determined by immunofluorescence and immunoblot analysis. The further development towards migratory neural crest was associated with decreased CD24, CD49f (ITGA6) and CD57 (HNK1) versus an enhanced CD49d (ITGA4), CD49e (ITGA5) and CD51/CD61 (ITGAV/ITGB3) expression. Notably, a shift from CD57 to CD51/CD61 was identified as a sensitive surrogate surface indicator of EMT in neural crest *in vitro* development. The reported changes in glycan epitope and integrin surface expression may prove useful for elucidating neural crest stemness, EMT progression and malignancies.

**Graphical Abstract:**

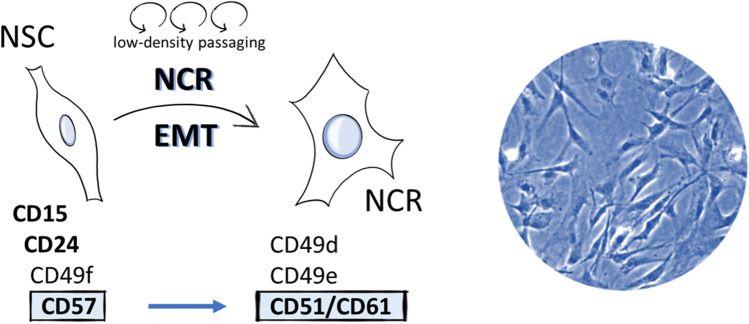

**Supplementary information:**

The online version contains supplementary material available at 10.1007/s12015-022-10393-1.

## Introduction

The neural crest (NCR) represents a unique transient population of cells specific to vertebrate ontogenesis [1–4]. Thought to arise from the neural plate border during neural tube closure, it comprises a transient multipotent population of developmental progenitor cells [1, 2]. In animal model systems, pre-migratory NCR cells transiently reside within the dorsal neural tube, then undergo epithelial-to-mesenchymal transition (EMT) and migrate toward various regions throughout the embryo. NCR derivatives include the peripheral nervous system, craniofacial skeleton, melanocytes, Schwann cells and the enteric nervous system, making NCR cells a particularly attractive and relevant candidate source for applications in regenerative medicine [5–8]. While an isolation of early primary NCR cells from the endogenous environment in humans is hampered by technical as well as ethical issues [9–11], pluripotent stem cell (PSC) systems may provide a prolific and standardizable source for human NCR cells [2, 12–14]. Moreover, PSCs offer *in vitro* tools to investigate these otherwise difficult-to-study early developmental processes in a human model. *In vitro* culture systems from human PSCs aim to mimic developmental inputs to generate/induce neural cell progeny [15]. Several protocols have become available to generate NCR cells from human PSCs (for review, see [2]). However, most of these either utilize incompletely defined culture conditions [16–19] or growth as non-adherent cultures and complex cell sorting steps [20–23]. For therapeutic applicability, NCR cell preparations and other NCR-derived advanced therapy medicinal products (ATMPs) will have to be well defined, standardized and safe for clinical use [13, 14]. Such protocols would greatly profit from a clear definition of NCR differentiation stages according to characteristic, phenotype-specific markers quantifiable in live cells [24–26]. Accordingly, a chemically defined method has recently been developed to generate NCR [27]. A better characterization of NCR cell stages will be equally critical for developing clinical cell transplantation paradigms [28, 29], as well as for utilizing *in vitro* models of NCR-related human disease [30]. Beyond transcriptomic marker analysis [4, 31], surface markers have not yet systematically been characterized or reported. Empirically, since early attempts at human PSC NCR differentiation, surface epitopes have been used to enrich NCR cells, e.g. via fluorescence-activated cell sorting (FACS), from the otherwise heterogeneous differentiation protocols [32] or to separate these from murine stromal co-culture layers and other cellular contaminants. To characterize hematopoietic [33] or mesenchymal stem cell therapeutics [34], rather established and extensive panels of CD surface molecules are routinely applied in clinical cell therapy applications as selection and/or quality control (QC) markers. For NCR(-like) stem cells, the markers applied have been rather simplistic and comparably not well-resolved: most commonly, as also used in classic NCR animal models, the low-affinity nerve growth receptor (LNGFR; neurotrophin receptor p75) CD271 antigen has been utilized as a NCR surface marker in human pluripotent stem cell-derived NCR cell preparations [17, 32] – disregarding its overall limited specificity due to co-expression on neural progenitor cells [35], mature neurons [36–38] as well as mesenchymal stem cells [39, 40]. In conjunction with CD271, the CD90 epitope (THY-1) has been used in NC characterization or isolation, while also being expressed on neurons [38, 41] as well as mesenchymal stem cells. As another commonly used label, the human natural killer-1 (HNK1) epitope (CD57; LEU7) is known to be expressed in human, rat and chick (but supposedly not mouse) NCR [42, 43]. However, CD57 is also expressed on striated muscle [44], natural killer (NK) and T-cell subsets [45], underlining how additional markers and marker combinations are required to uniquely identify and better characterize NCR differentiation stages. For example, in PSC cultures β1-integrin (ITGB1, CD29) has been found to be highly expressed in NCR-like “contaminants” in central nervous system-targeting neuronal cell therapeutic preparations [24]. In line with these observations, CD29 as well as the α4-integrin subunit epitope CD49d (ITGA4) have been found to be highly expressed upon initiation of human PSC-derived NCR differentiation [46]. More recently, we identified the transferrin receptor-1 (TFR1, CD71) as an exploitable candidate also for the elimination of NCR cells from human PSC-derived neuronal cell preparations in the differentiation paradigm applied, however, with limited specificity due to its almost ubiquitous expression on other non-neuronal cell types [38]. These data illustrate that a more systematic analysis of NCR marker expression dynamics and combinatorial expression patterns may be warranted. Here, we set out to characterize NCR cells derived from human PSCs according to cluster-of-differentiation (CD) antigens. Based on previous work [17, 46] we share an easy-to-implement, defined feeder-free protocol for the derivation of NCR cells from human embryonic and induced pluripotent stem cells, largely via modulating cellular plating densities. Moreover, we report a detailed signature of cluster-of-differentiation (CD) markers associated with NCR induction, neural EMT and acquisition of mesenchymal features, which will facilitate NCR stage-specific monitoring, quality control and quantitative analysis and may thereby aid in devising clinical-grade NCR cell preparations.

## Materials and Methods

### Human PSC Propagation

H9 (WA-09) human embryonic stem cells were obtained from WiCell (Madison, WI) and, after initial feeder-based expansion on a layer of mitotically inactive human fibroblasts (D551), routinely cultured under feeder-free conditions on Synthemax II-SC substrate (Corning)-coated cell culture plates (as described previously [46]).

### Human PSC Differentiation to Neuronal and Neural Crest Lineage

For neural induction, H9 embryonic stem cells were harvested and plated (at 0 days *in vitro*; DIV0) onto Synthemax II-SC (Corning) coated wells at a density of 200,000 cells/cm^2^ in E8 medium supplemented with 10 µM Y-27,632 (Sigma). Neural induction was initiated on DIV1 by Neural Induction Medium 1 (NIM1) consisting of E8 medium (Thermo Fisher Scientific) supplemented with 10 µM SB431542 (Tocris) and 1 µM Dorsomorphin (Tocris). Cells were maintained in NIM1 (100%) with daily media changes for a total of 5 days. On DIV6, medium was changed to a 3:1 (part-to-part ratio) combination of NIM1 (75%) and NIM2 (25%; comprising DMEM/F-12 supplemented with 1x N2 supplement, 10 µM SB431542 and 1 µM Dorsomorphin). On DIV7 and DIV8, media were changed to a 1:1 (50/50%) and 1:3 (25/75%) ratio of NIM1:NIM2 respectively. From DIV9 until the end of neural induction (DIV12), the cells were maintained in NIM2 medium (100%) with daily media changes. The resulting high-density neural stem cells (NSCs) were then harvested and plated onto PO/laminin-coated wells in NSC medium comprising DMEM/F-12 supplemented with N2 supplement, 20 ng/ml FGF-2, 20 ng/ml EGF (Peprotech) for maintenance and (for subsequent differentiation towards central nervous system-type neurons or) to direct them towards NCR lineage. The NSCs generated at DIV12 were plated at a high-density (500,000 cells/cm^2^) for expansion. These NSCs were passaged for up to four times. For neuronal differentiation, P4 NSCs were plated at 200,000 cells/cm^2^ in differentiation medium comprising Neurobasal medium with 1% B27 supplement (both Thermo Fisher Scientific), 20 ng/ml Brain derived neurotrophic factor (BDNF, Peprotech), 20 ng/ml GDNF, 200 µM Ascorbic acid (Sigma), 0.5 mM cAMP and 2mM L-glutamine (adapted and modified from [47]) for 14 days. For differentiation to NCR lineage, the NSCs at DIV12 were plated at low-density (20,000 cells/cm^2^) condition and passaged as such for up to nine passages in NSC medium. 2 µM Thiazovivin (Selleck Chemicals) was added to the culture medium at each passaging step.

### Differentiation to Peripheral Neurons, Adipocytes, Chondrocytes and Osteocytes

For peripheral neural differentiation, early to mid-stage NCR cells (P2 – P5) were plated on PO/Laminin-coated cover slips at 200,000 cells/cm^2^ and treated for 14 days with differentiation media DMEM/F-12 supplemented with N2 supplement, 10 ng/ml BDNF, 200 µM Ascorbic acid, 10 ng/ml GDNF, 10 ng/ml Nerve growth factor (Peprotech), 10 ng/ml Neurotrophin 3 (Peprotech) and 0.5 mM cAMP (adapted from [21]). Fresh medium was added to the cells every other day. Late-stage NCR cultures (P6 – P9) were plated on 0.1% gelatin-coated plates for adipocyte (10,000 cells/cm^2^), osteocyte (5000 cells/cm^2^) and chondrocyte (droplet plated with 80,000 cells/ 10 µl droplet) culture. Differentiation was carried out using the StemPro Adipogenesis, Osteogenesis and Chondrogenesis (Thermo Fisher Scientific) kits according to the manufacturer’s instructions. Cultures were stained after 3 to 4 weeks using oil red O, alizarin red S and alcian blue for the presence of adipocytes, osteocytes and chondrocytes, respectively.

### Immunoblot Analysis

Harvesting for experimental analysis was done by enzymatic digestion using TrypLE Express (Thermo Fisher Scientific) and centrifugation at 2000* g*, followed by a wash with 1x PBS (without Ca^++^ and Mg^++^, Thermo Fisher Scientific). The resulting pellet was stored at -20 °C or -80 °C for immunoblotting. Cell pellets were lyzed using 3x Sample buffer made up of 1 M Tris HCl (pH 6.8), 15% SDS and 15% Glycerol, boiled at 95**°**C for 10 min and centrifuged for 10 min. Proteins were size-separated using SDS gel electrophoresis (Mini-PROTEAN TGX precast gels, 4–20% gradient, Biorad) followed by transfer to a PVDF membrane (Merck Millipore). Membranes were incubated for 1 h at room temperature in blocking buffer made up of 4% skimmed milk powder (Fluka) in PBS with 0.2% Tween-20 (AppliChem) followed by incubation with primary antibody diluted in blocking buffer overnight at 4**°**C. Unbound antibody was washed off using 3 washes with PBST (PBS with 0.2% Tween-20), followed by incubation with HRP-conjugated secondary antibodies for 1 h at room temperature and three washes. Membranes were developed using Amersham ECL Prime western blotting detection reagent (GE Healthcare) or WesternBright Sirius HRP substrate (Advansta) and chemiluminiscence was imaged using ImageQuant LAS 4000 (GE Healthcare) and bands were quantified by ImageJ software. Details of antibodies used are given in Table 1.

**Table 1:** List of antibodies used for immunocytochemistry (ICC) and immunoblotting (IB/WB)

Antibody	Supplier	Host	Cat. No:	Dilution	
				ICC	IB/WB
AP2-α	Abcam	Rabbit	ab52222		1:500
BRN3A	Millipore	Mouse	AB5945	1:200	
CD29	BD Pharmingen	Rat	552,828		1:200
CD44 FITC	E Bioscience	Rat	11-044186	1:50	
CD49d	Cell Signaling	Rabbit	8440		1:1000
Doublecortin	Santa Cruz	Goat	sc-8066	1:200	1:500
HNK1	Santa Cruz	Mouse	sc-81,633		1:200
HNK1/CD57	BD Bioscience	Mouse	559,048	1:100	
MAP2	Millipore	Mouse	MAB3418		1:1000
Slug	Cell Signaling	Rabbit	9585		1:500
SNAIL	Cell Signaling	Rabbit	3879		1:500
SOX2	R&D Systems	Goat	AF2018	1:500	1:1000
βTubulin	Abcam	Rabbit	ab6046		1:2000
Twist-1	Abcam	Mouse	ab50887		1:50
Vimentin	BD Pharmingen	Mouse	550,513	1:1000	1:10000
ZEB1	Cell Signaling	Rabbit	3396		1:500

### Immunocytochemistry

Cells were fixed using 4% paraformaldehyde solution for 30 min, followed by permeabilization in 0.5% Triton X-100 (Sigma) solution for 10 min, and blocking in Ca^++^/Mg^++^-free DPBS-buffered blocking solution containing 10% normal donkey serum (Millipore) and 1% bovine serum albumin for 30 min. Cells were then incubated with primary antibody diluted in blocking solution overnight at 4**°**C. Cells were washed 3 times using DPBS, followed by incubation with secondary antibody (1:500 in blocking solution, Alexa Fluor-488 and − 568 conjugated secondary antibodies; Thermo Fisher Scientific) for 30 min at room temperature. Nuclei were counterstained using Hoechst (1:10,000, Thermo Fisher Scientific) added to the secondary antibody solution. Stained cells were washed and mounted onto glass slides using ProLong Gold or ProLong Diamond (both Thermo Fisher Scientific) antifade mountant. Images were acquired using AxioImager-M2 and AxioPlan-2 fluorescence microscopes and analyzed using Zen Blue software (Zeiss). Details and concentrations of all primary antibodies used for immunocytochemistry are given in Table 1.

### Flow Cytometric Analysis

Cells harvested using TrypLE were re-suspended to a concentration of 0.5 to 2 × 10^6^ cells/ml in flow cytometry (FC) buffer made up of DPBS (Ca^++^ and Mg^++^-free, Gibco) and 2% FBS [48–50]. 100 µl of cell suspension was used for each staining sample and 2 µl of fluorophore-conjugated primary CD antibodies were added prior to 30 min incubation on a shaker protected from light. After 3 washes using FC buffer for 3 min at 376 g on a centrifuge maintained at 4**°**C, the resulting pellet was re-suspended in 100 µl of FC buffer and analyzed using a BD Accuri C6 (BD Biosciences) benchtop flow cytometer and corresponding software. Details on antibodies used for flow cytometry are given in Table 2. Live population gates, doublet exclusion and determination of percent positivity was done as described before [48, 49]. Co-expression and Spearman correlation was performed via a combined and integrated analysis across NCR differentiation, including P0, P3, P6, P9 as well as P3, P4, P5, P6 experimental series.

**Table 2:** List of antibodies used for flow cytometry

Antibody	Manufacturer	Clone/Isotype	Cat. No.	Dilution
CD15 FITC	eBioscience	HI98	11-0159-42	1:50
CD24 APC	eBioscience	SN3 A5-2H10	17-0247-42	1:50
CD29 FITC	eBioscience	TS2/16	11-0299-42	1:50
CD29 PE	eBioscience	TS2/16	12-0299-42	1:50
CD29 APC	eBioscience	TS2/16	17-0299-42	1:50
CD44 FITC	eBioscience	IM7	11-0441-86	1:50
CD49d PE	eBioscience	9F10	12-0499-42	1:50
CD49e Alexa Fluor 488	eBioscience	SAM-1	53-0496-41	1:50
CD146 PE	eBioscience	P1H12	12-1469-41	1:50
CD49f PE	eBioscience	GoH3	12-0495-83	1:50
CD51/61 FITC	eBioscience	23C6	11-0519-42	1:50
CD57 FITC	eBioscience	TB01(TBO1)	11-0577-42	1:50
CD73 PE	eBioscience	AD2	12-0739-42	1:50
CD90 APC	eBioscience	5E10	17-0909-42	1:50
CD133 APC	MACS	AC133	130-090-826	1:50
CD200 APC	eBioscience	OX104	17-9200-42	1:50
CD271 Alexa Fluor 647	BD Pharmingen	C40-1457	560,326	1:50

## Results

### Generation of Neural Crest Cells from Human Pluripotent Stem Cells

**Fig. 1:**
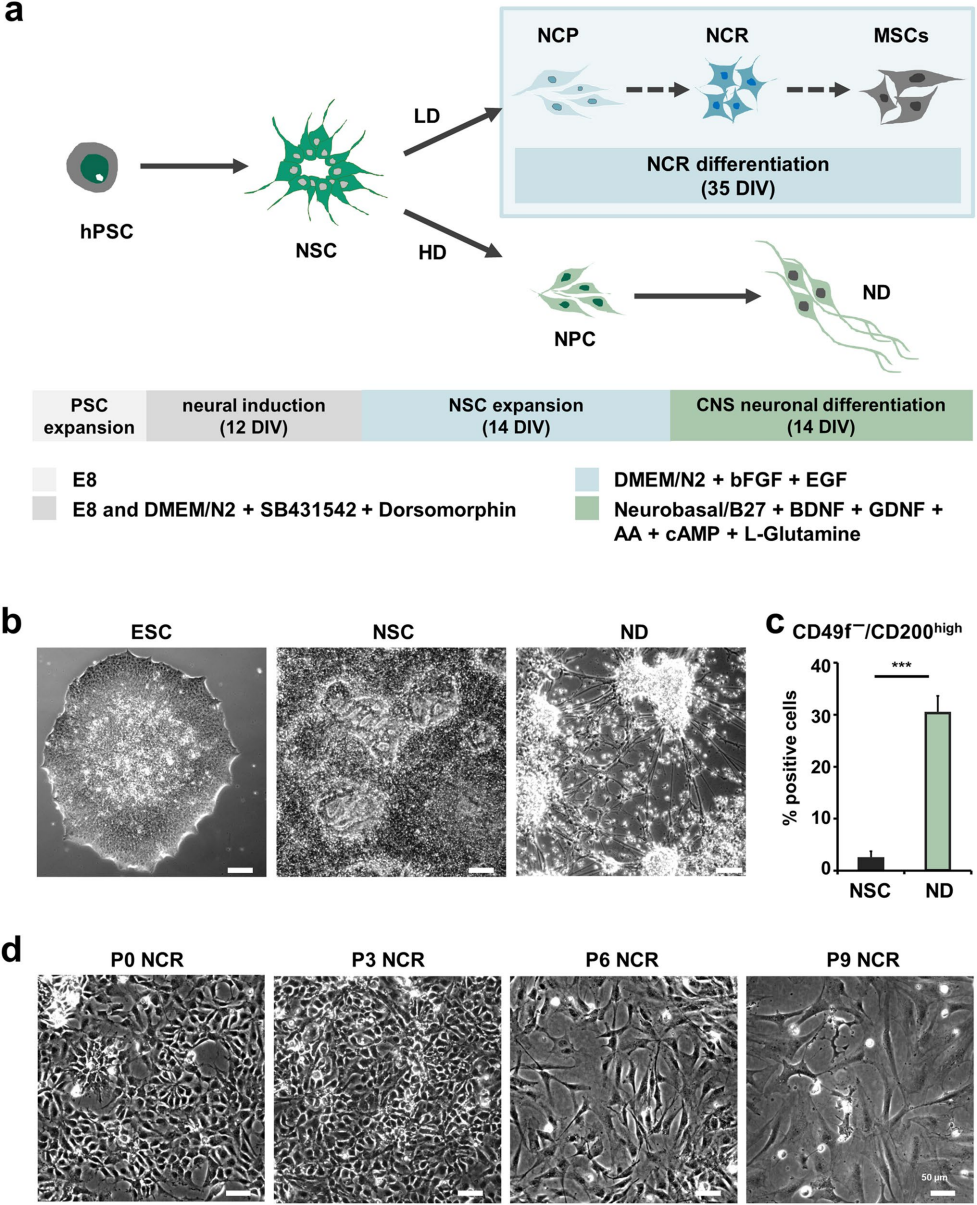
Generation of neural crest cells from human embryonic stem cell-derived neural progenitors. (**a**) Schematic outlining the steps for generation of neuronal (ND) and different stages of neural crest (NCR) progeny (NCR precursors, NCPs; migratory NCR; increasingly mesenchymal stem cell-like cells, MSCs) from hPSCs. (**b**) Phase contrast images of hESCs (H9), NSCs (P0) and neurons (ND) after 14 days of differentiation, depicting morphology of the respective stages (Scale bars: 100 μm). (**c**) Flow cytometric analysis revealing the presence of a population exhibiting the surface marker code CD49f^−^ /200^+^ underlining that the neuronal fraction is significantly enriched in ND compared to NSCs (also see [48] and Suppl. Fig. 1). (**d**) Phase contrast images of various passages of NCR differentiation, illustrating the morphology of P0-P2 NCR, P3-P5 NCR and late stage (P6-P9) mesenchymal passaging stages (Scale bars: 50 μm)

We developed and employed an easy-to-use, fully defined protocol (Fig. 1a) for the generation of NCR cells versus central nervous system neurons from a common rosette forming neural stem/ progenitor cell pool primarily via manipulating the cellular plating density. High-density (200,000 cells/cm^2^) neuronal differentiation conditions resulted in the sequential acquisition of typical morphological features from pluripotent cell colonies to polarized neuroepithelial stem cells capable of forming rosette-like structures and towards process-bearing neurons (Fig. 1b). Comparing the surface marker profile of NSCs (passage 1) versus 14 days of neuronal differentiation, we observed a downregulation in markers associated with proliferation and neural stemness (CD15, CD49f, CD184) and an upregulation of markers associated with neuronal phenotype (CD24 and CD200^high^), in keeping with prior studies. The efficacy of neuronal differentiation was most notably underlined by the significant enrichment (11.8-fold) of the neuronal subset defined by the known surface marker code CD49f^−^/200^+^ in neuronal differentiation (ND) compared to NSCs (Fig. 1c and Suppl. Fig. 1). In contrast, using the same original neuroepithelial progenitor pool as starting material, upon adjusting cell plating densities to ten-fold lower levels (20,000 cells/cm^2^), phenotypic changes towards increasingly mesenchymal morphology with high cytoplasmic-nuclear ratio were observed over the course of propagation and passaging time *in vitro* (Fig. 1d). Through consecutive rounds of passaging, three distinct stages could be morphologically categorized. Cells at early low-density passages (P0 – P2) were smaller with little cytoplasmic rim and partially still polarized morphology. Mid passages (P3 – P5) showed larger spreading, flat cell NCR morphology, while late stage cells (P6 – P9) displayed increasingly mesenchymal phenotypes.

### Neural Crest Stage Phenotypic Characterization and Multipotency

**Fig. 2:**
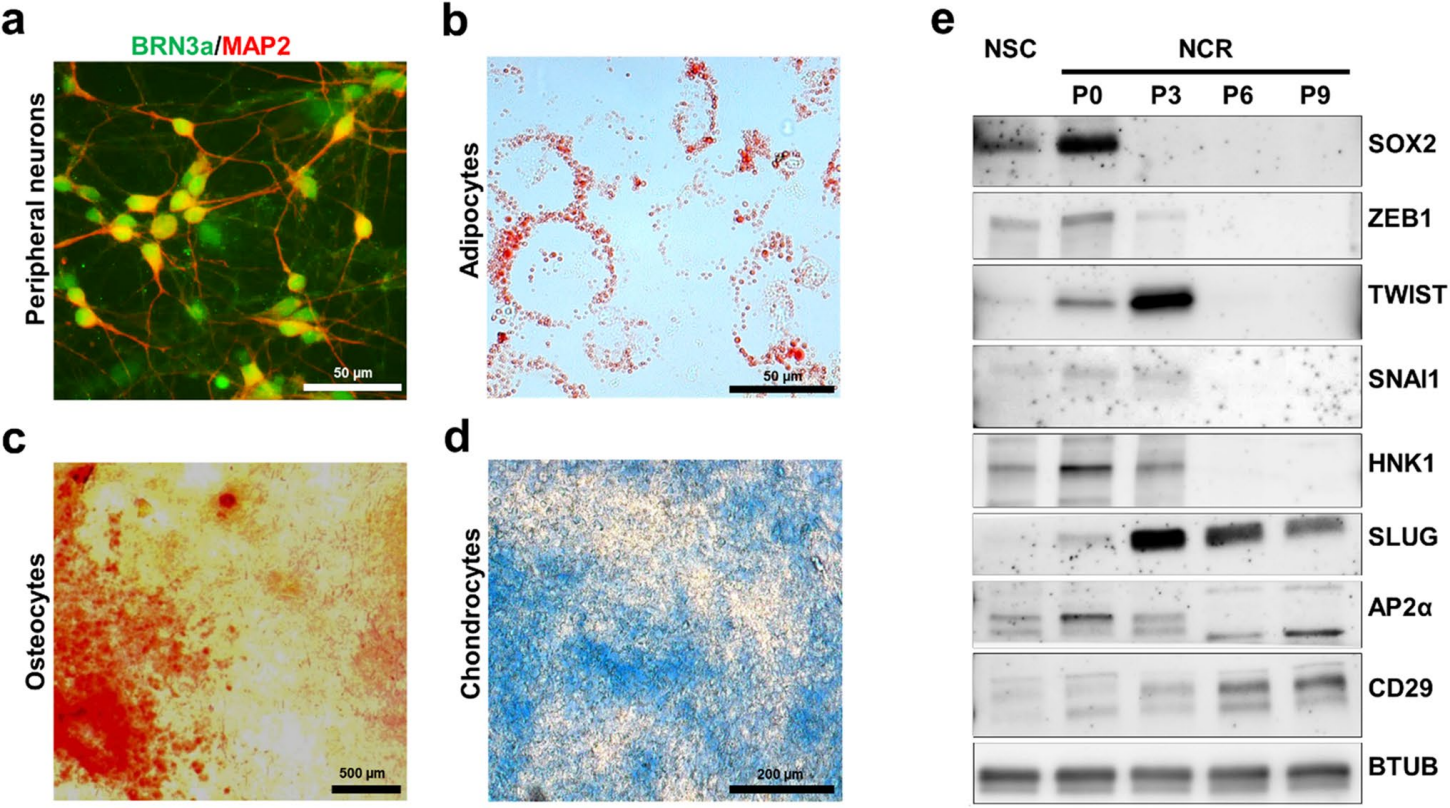
Protein expression changes and epithelial-to-mesenchymal (EMT) markers in neural crest differentiation. (**a**) Multipotency of generated NCR cells (P5 to P9) was confirmed by its ability to differentiate towards peripheral neuronal lineage (co-expressing BRN3A and MAP2), as well as into (**b**) adipocyte (stained positive for oil red O), (**c**) osteocyte (stained positive for alizarin red) and (**d**) chondrocyte phenotypes (stained positive for alcian blue). (**e**) Immunoblot analysis comparing the expression of neural stemness (SOX2, ZEB1), EMT (TWIST1, SNAI1, SLUG/SNAI2, HNK1) and NCR and MSC-related proteins (ITGB1/CD29; TFAP2A/AP2α) at neural stem cell (NSC) stage and across different passages of NCR cell culture (NCR P0 to P9)

NCR (P3 – P5) and mesenchymal stem cell-like (P6 – P9) phenotypes generated in this low density-based protocol were differentiated into brain-specific homeobox/POU domain protein 3 A (BRN3A) and microtubule-associated protein-2 (MAP2)-positive peripheral neurons (Fig. 2a), oil red O-positive adipocytes (Fig. 2b), alizarin red-positive osteocytes (Fig. 2c) and alcian blue-positive chondrocytes (Fig. 2d), thus confirming NCR multipotency and lineage differentiation potential. Protein expression analysis by immunoblot (Fig. 2e) showed the expression of neuroepithelium-associated SOX2 and the *bona fide* EMT activator ZEB1 at P0, thus consistent with pre-migratory neural phenotypes. EMT-associated TWIST1, SNAI1, SLUG (SNAI2) and migratory NCR-associated HNK1 (CD57) were most closely associated with P3, while the post-migratory marker TFAP2A (AP2-α) and the NCR and mesenchymal marker CD29 (ITGB1) were strongly upregulated over the course of passages P6 and P9, thus confirming the sequential presence of pre-migratory (P0 – P2), EMT/ early migratory NCR (P3 – P5) and increasingly mesenchymal stem cell-like (P6-P9) populations during the course of differentiation in low-density culture conditions.

**Fig. 3:**
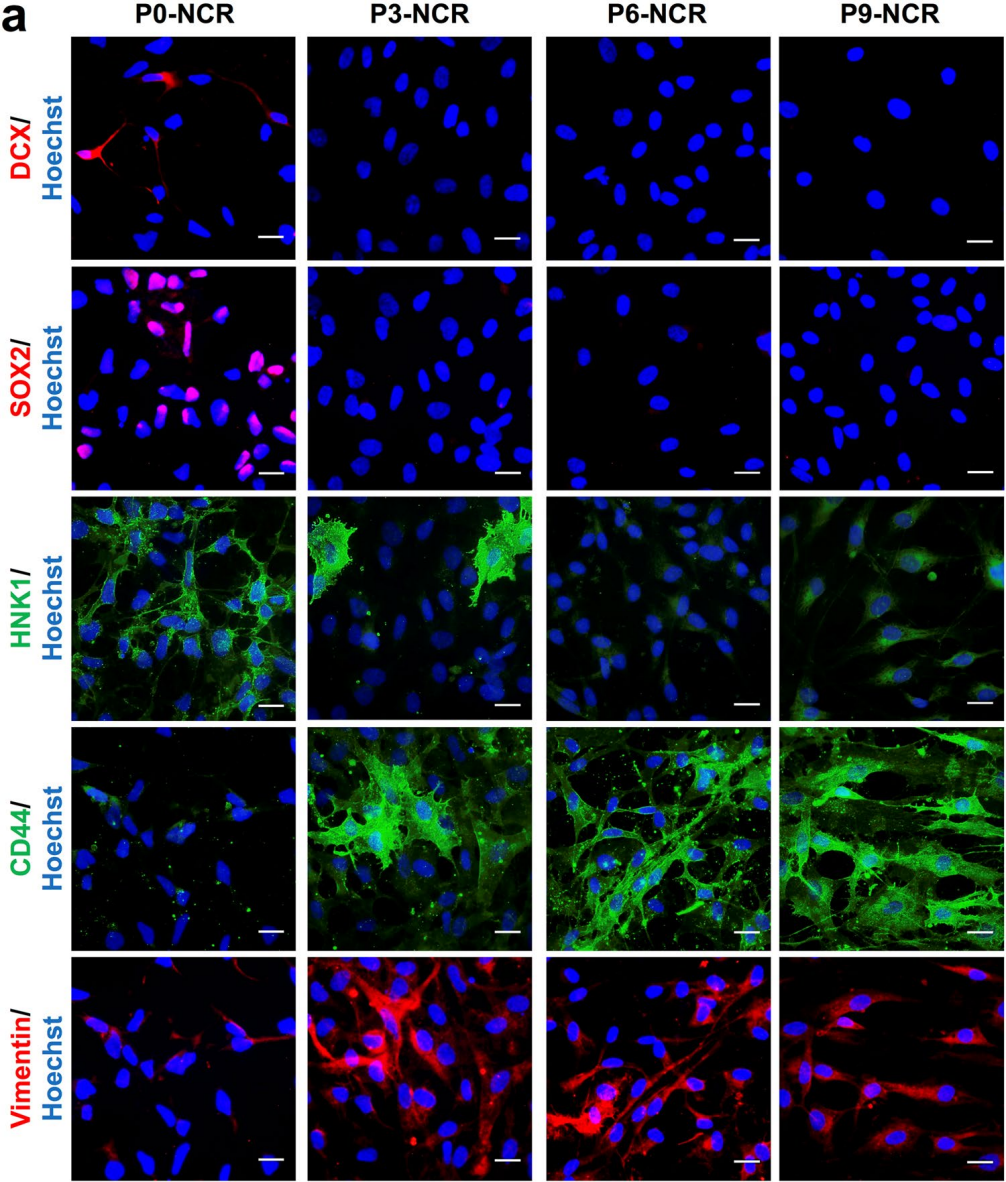
Lineage marker analysis confirms the presence of distinct developmental stages in long-term neural crest culture. (**a**) Immunocytochemical analysis of different passages throughout NCR differentiation (P0, P3, P6 and P9) for neural progenitors (DCX and SOX2), NCR (HNK1/CD57) and mesenchymal cells (CD44, Vimentin) confirms the presence of clearly distinct phenotypic stages in long-term NCR culture in congruence with morphological changes (Scale bar: 50 μm)

In agreement with the immunoblot data, the microscopical protein expression analyses of these cells using immunocytochemistry (Fig. 3a) for doublecortin, SOX2, HNK1, CD44 and vimentin confirmed the sequential changes of phenotypes from still polarized, stem cell marker-bearing NCR precursors towards mesenchymal phenotypes over time *in vitro* (P0 – P9). Clearly, P3 stood out as a transitional stage, where NSC-like HNK1 expression was jointly present with early CD44 and vimentin, while SOX2 and doublecortin were clearly already downregulated. Based on morphological and protein expression data (see Figs. 1, 2 and 3), passages P3 to P5 were designated as the stages most closely representing neuroepithelial-to-mesenchymal transitions in this protocol. To better understand these stages at the level of surface marker signatures enabling quantitative live-cell monitoring of intermediate developmental stages in culture, we next performed a flow cytometric analyses of the various passages of NCR cell cultures (P0, P3, P6 and P9) using stage-specific cluster of differentiation (CD) markers.

### Surface Marker Profiles of Neural Crest Differentiation

**Fig. 4:**
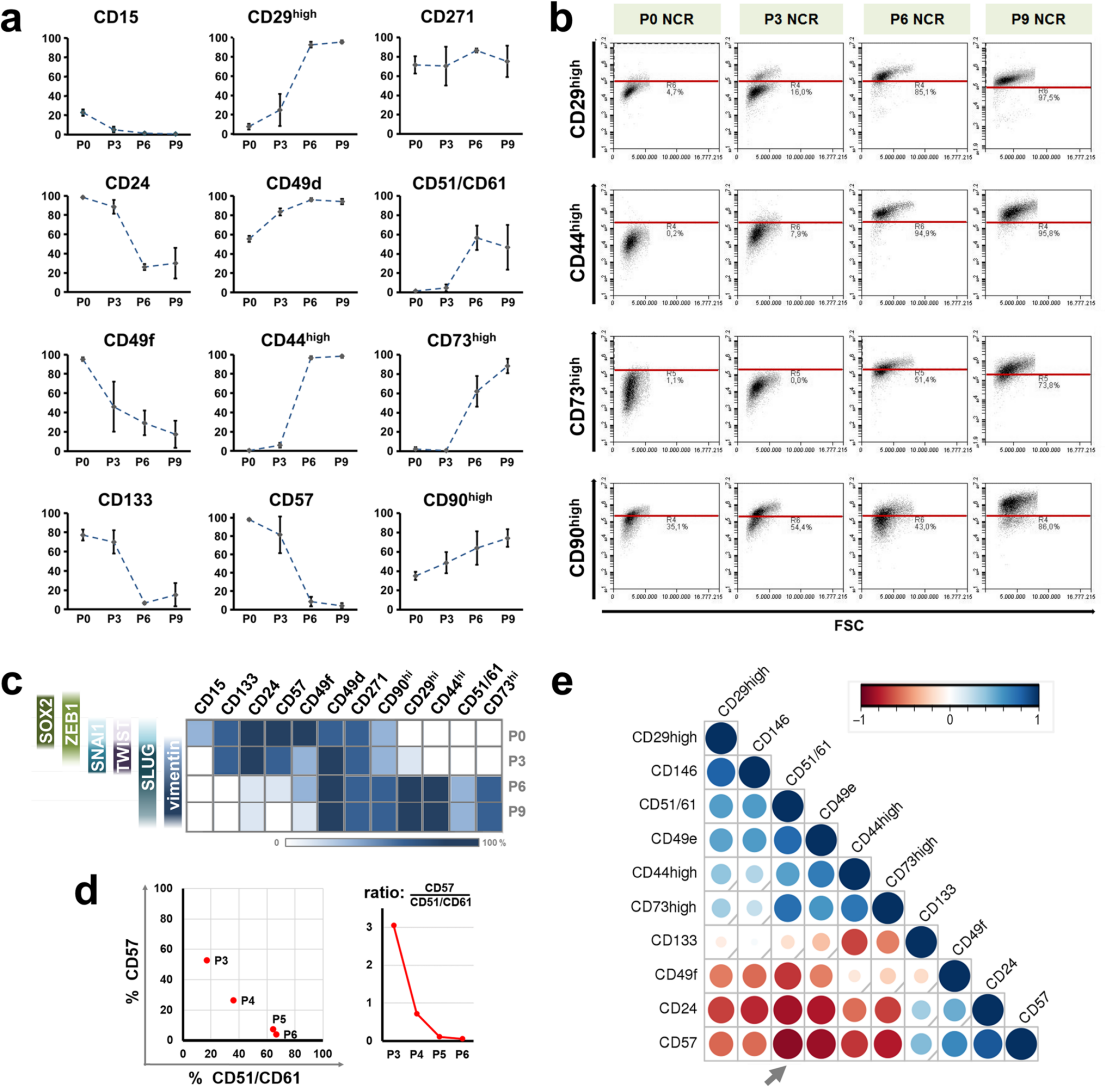
Surface marker expression changes in PSC-derived neural crest development. (**a**) Flow cytometric characterization of surface markers across four different stages of NCR differentiation (Passages 0, 3, 6 and 9) shows the temporal changes in surface marker expression as the cells progress along NCR differentiation. Error bars represent arithmetic mean ± SD (n = 3; CD90^high^ at P9 n = 2). (**b**) Figure depicts the arbitrary threshold (indicated by red gate line) set and consistently applied across the various passages of NCR differentiation to identify CD29d^high^, CD44^high^, CD73^high^ and CD90^high^ cells. (**c**) Heatmap representation of CD antigen levels (percentage of expression) in relation to established intracellular NCR and EMT protein expression (lines of squares indicating passage no. at P0, P3, P6, P9; also see Fig. 2e and Suppl. Fig. 2), illustrating a critical transition stage around P3 and association of CD markers with either NSC (left, top) or post-EMT (right, bottom) phenotypes. (**d**) Time-dependent correlation of percentage of CD57 versus CD51/CD61 expression underlining a gradual decrease of HNK1 accompanied by a gain of CD51/61 expression when focusing on P3 to P6 as a critical transition period (left panel), with the clearest drop occuring between P3 to P4 (right panel). (**e**) Spearman correlation matrix of flow cytometric expression data across NCR differentiation (percentage positivity at P0, P3, P4, P5, P6, P9) highlights the largest negative correlation of surface antigen expression of CD57 vs. CD51/61 (–0.89; see Suppl. Table 1) over the course of NCR EMT

In accordance with categories identified by morphology and analysis based on established marker proteins by immunoblot and immunofluorescence, flow cytometric analyses revealed three clearly distinct stages in terms of CD molecule expression patterns (Fig. 4a, b). Over the course of early passages (P0 – P3) markers associated with stemness and neuronal phenotype were progressively downregulated. Specifically, CD15 (Lewis-X antigen, SSEA-1), a known neuroepithelial marker decreased from 22.7 ± 3.1% at P0 to close to 5.1 ± 2.8% in P3 NCR (while expressed at ca. 60% in NSCs also see Suppl. Fig. 1). Similarly, CD49f (ITGA6), previously identifed by our group on PSC-derived NSCs was reduced from almost ubiquitous expression at P0 (> 95.3 ± 1.9%) to ca. 45.9 ± 26.0% at P3. In contrast, CD133 (prominin-1) a widely used stem cell marker in various tissues throughout the body including NCR-derived tumors remained highly expressed without significant reduction from P0 to P3 (77.1 ± 5.6% to 70.1 ± 12.2%, respectively). Notably, CD271 (p75/LNGFR) as a rather commonly used marker of NCR phenotype was consistently expressed at stable levels throughout NCR *in vitro* expansion, without significant change. CD24, present on NSCs and neurons similarly continued to be expressed on the cell surface, as was the established putative NCR marker HNK1 (CD57) at this stage, prior to a considerable downregulation from P3 to P6. In contrast to these previous glycosylated epitopes, CD49d and CD51/CD61 surface antigens were significantly upregulated over the course of NCR differentiation. In addition to overall percentual changes, a number of surface molecules enhanced per-cell levels of expression over the course of the later passages *in vitro*, reflected by the increased presence of the CD29^high^, CD44^high^, CD73^high^ and CD90^high^ populations (enhanced mean fluorescence intensity; Fig. 4b).

By surface marker analysis, NCR EMT could thus be defined by the respective expression patterns of initially losing neural stemness (CD15, CD49f, CD133). Subsequently, more profoundly, pre-migratory neuroepithelial features defined by other glycan epitopes (CD24, CD57) were lost, followed by enhanced expression of migration-promoting integrin subunits (CD29, CD49d, CD51/CD61) and, eventually, increasingly mesenchymal surface molecular features (CD44, CD90; CD73^high^; see Fig. 4c). Additional CD expression studies at a greater temporal resolution of the critical P3 to P6 period confirmed a shift from glycan epitopes such as CD24 and to CD57 towards CD49e and CD51/CD61 integrins (Fig. 4c-e and Suppl. Fig. 2). Finally, an integrated correlation analysis across NCR differentiation (including P0, P3, P4, P5, P6, P9 data points) showed that the loss of CD57 and occurrence of CD51/CD61 was the marker shift most closely associated with and most sensitive for detecting the phenotypic transition occurring at this stage (NCR EMT): identifying clearly distinct clusters in bivariate expression analysis of these markers over time (Fig. 4d), and also showing the clearest expression disparity in NCR development across all passages (Spearman correlation coefficient of − 0.89; Fig. 4e, see Suppl. Table 1).

## Discussion

The ability of human PSCs to differentiate to derivatives of all three germ layers has proven to be a valuable tool for elucidating human neural development and for modeling diseases *in vitro* [51, 52]. Here, we report a simplified, scalable protocol for the generation of NCR lineage from human PSC-derived neural progenitors, based on our understanding of the cell surface-mediated biological processes that modulate human neural development [2, 4, 46, 53]. Previous studies described the generation of neuroepithelial precursors from human PSCs via dual SMAD inhibition: the synergistic action of the two small molecules noggin and SB431542 have long been known to produce mostly PAX6^+^ neuroectodermal progenitors with high efficiency in adherent culture conditions. At low cell density, however, the same neural induction conditions were reported to result in a mixture of PAX6^+^ and p75^+^/HNK1^+^ NCR cells [54]. In line with this, another report showed that the plating density of PSA-NCAM^+^ neural precursor cells determined their differentiation propensity, with lower density favoring cells of NCR character [22]. Further, our own research implicated the cell density-dependent Hippo/YAP-TAZ signaling pathway as a critical regulator of NCR development [46]. In the protocol applied here, we show that rosette-forming neuroepithelial precursors can be successfully maintained as NSCs and subsequently differentiated to neurons when plated at a high density (200,000 cells/cm^2^). On the other hand, at low density (20,000 cells/cm^2^), they may be efficiently differentiated to various stages of NCR lineage (see [46]). In addition to cost-efficiency and scalability, a key feature of this current protocol is that the cells generated can emulate various stages of NCR development. Upon repeated passaging at low density conditions, three distinct sequential phases could be discerned – an initial pre-migratory neuroepithelial NCR precursor stage (exhibiting SOX1, ZEB1; P0 – P2); an EMT-phase NCR stage with migratory properties and positivity for HNK1, SNAI1, SLUG/SNAI2 and TWIST (P3 – P5); and a later migratory mesenchymal stem cell-like stage positive for CD73, CD90, CD44, CD49d, CD29 and CD51/61 (P6 – P9).

In congruence with our previous work [24, 48] and other prior studies [55, 56] P0 NCR cells still bear neuroepithelial markers (CD15, CD49f, CD133 [57, 58]) on their surface as well as by immunocytochemical analysis (e.g., SOX2, ZEB1). Other groups have shown that SOX2 is a hallmark of pre-migratory NCR cells [59] and that SOX2 downregulation is sufficient to induce and control EMT. Moreover, ZEB1 expression may be associated with maintaining neural stemness [60, 61]. On the other hand, low levels of p75/CD271 [21, 62] and HNK1/CD57 are also expressed here, in conjunction with ZEB1/TWIST1/AP2A and thereby already clearly distinct from NSC phenotype. In comparison to the preceding NSC stage, according to surface markers, this stage (P0 NCR) is characterized by maintained expression of stemness markers CD49f and CD133, the *bona fide* pan-neural marker CD24 as well as low-level positivity for CD90 paired with increasing levels of CD57 and CD49d. Notably, CD133 (a stem cell marker in various tissue systems including neural stem cells [58]) has previously been used to isolate stem cells of NCR origin [55]. Thus, in the stem cell system studied here, P0 NCR, represents an early pre-migratory NCR precursor stage that is distinguishable from NSC and later NCR stages (beyond P3) by co-expression of CD24, CD49f and CD133 with a combinatorial expression of CD57. The clear morphological and overall phenotypic change occurring with P3 NCR is accompanied by acquisition of more prominent vimentin and CD44 expression as well as a profound upregulation of the known EMT activators ZEB1, TWIST and SLUG [59, 61, 63, 64]. While overall CD44 expression is clearly increased, an excessive CD44 expression per cell (CD44^high^) has not yet been reached at this stage. In addition to own prior work [24], CD44 has recently been implicated as a marker for NCR stem-like cells [65]. Positivity for CD24, CD49d and negativity for CD49f and CD15 characterize this NCR transitional stage (pre-migratory to EMT; also see [46]). As is also the case *in vivo*, SNAI1 expression (P0, P3) precedes CD51/CD61 induction (at P6) [66].

The mesenchymal migratory NCR phenotype is consolidated over P6 to P9 NCR, reflected by loss of CD24, CD133 and CD57 and acquisition of CD29^high^ and CD44^high^ eventually across the entire cell population. Notably, also increased per-cell levels of CD73^high^ and CD90^high^ accompany this development as known mesenchymal stem cell markers [41, 67–70]. Late-passage NCR derivatives generated here were comparable to mesenchymal phenotypes obtained from NCR cells by repeated passaging in low density conditions in terms of morphology, surface marker profile and cell proliferation potential [41, 71]. We have previously observed a correlation of CD49d expression and nuclear localization of the Hippo pathway transcriptional regulator YAP in low-density human neural differentiation culture systems [46]. Similarly, loss of CD15 expression had previously been observed [24, 46]. Historically, others [17, 32] had characterized human NCR as p75^+^/HNK1^+^ (CD271^+^/CD57^+^) and we have previously seen CD15^−^/CD44^+^/CD49d^+^ as NCR signatures comprising both pre-migratory and migratory NCR sub-populations [46]. The results of this current study underline how NCR EMT can be identified, specifically and sensitively, by the surface expression shift from CD57 and other glycan moieties (CD15, CD24) towards a pro-migratory integrin panel of surface molecules including, most notably, CD51/61 (αVβ3). These results are corroborated by the alterations of glycan moities during epithelial-to-mesenchymal transition and neural development [72–75]. Our data show that TWIST and SLUG as established intracellular NCR markers are most closely associated with a population uniquely characterized by shifting from CD57 to CD51/CD61 at the cell surface level. CD51/CD61 seems to be regulated through complex signaling networks including the Hippo pathway downstream effector YAP [76]. Further, in the context of angiogenesis, the CCN1 protein, an upstream regulator of YAP was found to also enhance the expression of CD51/CD61 [77]. These results are in keeping with our findings of low-density culture conditions promoting YAP and its nuclear translocation in NCR [46]. While the direct signaling routes remain to be experimentally dissected, it is plausible that the reported low-density NCR differentiation protocol involves YAP as well as EMT-associated regulatory networks (ZEB1, TWIST, SNAI1, SLUG), thereby controlling a functionally relevant shift of glycan and integrin cell surface epitopes. The specific heterodimers most prominently upregulated at NCR EMT in the human stem cell system described here are congruent with the classically described involvement of primarily α4β1 and αvβ3 in cellular migration [78]. Beyond the inherent utility of stage-specific live-cell markers of human NCR EMT, the systematic, stage-specific analysis of CD surface antigen expression patterns of NCR *in vitro* development provides an enhanced resolution of NCR phenotype dynamics and transitions. Thereby the results obtained might prove useful not only for *in vitro* monitoring of NCR differentiation and quality control (QC) of NCR cell therapeutics, but also as histopathological markers in the context of NCR-associated tumors [79]. Interestingly, one of the most important proteins associated with melanoma potential is ITGB3/CD61 [80–82], and CD90 and CD51/CD61 have already been shown to functionally interact in melanoma cell adhesion [83].

In summary, this current analysis amends our and others NCR-associated marker codes (CD15/CD24/CD29 [24]; CD44/CD49d [46] with critical additions. Complementing established NCR transcriptional markers, the flow cytometrically assessable shift from CD57 epitopes towards CD51/CD61 surface expression further resolves the stages of neuroepithelial EMT. Such enhanced insights into NCR stemness and phenotypic regulation may contribute to more efficaciously realizing the broad therapeutic potential of human NCR *in vitro* models and stem cell preparations [2, 5].

## Supplementary Information

Below is the link to the electronic supplementary material.

ESM 1 (PDF 658 KB)

## Data Availability

The authors declare that all data supporting the findings of this study are available within the article (and its supplemental information) or from the corresponding author on reasonable request.
