# Supplementary material for: Glycan Epitope and Integrin Expression Dynamics Characterize Neural Crest Epithelial-to-Mesenchymal Transition (EMT) in Human Pluripotent Stem Cell Differentiation
Source: Stem Cell Rev Rep. 2022 Jun 21;18(8):2952–65. doi: 10.1007/s12015-022-10393-1 (PMC9622562; doi:10.1007/s12015-022-10393-1)
Supplement: Supplementary file 1 — (PDF 658 KB) [file 12015_2022_10393_MOESM1_ESM.pdf]

Supplementary Material

Thomas *et al*.:

Glycan epitope and integrin expression dynamics characterize neural crest epithelial-to-mesenchymal transition (EMT) in human pluripotent stem cell differentiation

Suppl. Table 1 – Spearman correlation matrix of CD antigens, as shown, across NCR *in vitro* culture.

marker	CD29^{high}	CD146	CD51/61	CD49e	CD44^{high}	CD73	CD133	CD49f	CD24	CD57
**CD29^{high}**	1									
**CD146**	0.8	1								
**CD51/61**	0.55	0.57	1							
**CD49e**	0.53	0.56	0.77	1						
**CD44^{high}**	0.39	0.29	0.54	0.69	1					
**CD73^{high}**	0.34	0.23	0.75	0.58	0.72	1				
**CD133**	-0.1	0.03	-0.17	-0.3	-0.67	-0.51	1			
**CD49f**	-0.52	-0.56	-0.72	-0.51	-0.13	-0.24	-0.19	1		
**CD24**	-0.69	-0.76	-0.84	-0.8	-0.55	-0.68	0.34	0.49	1	
**CD57**	-0.58	-0.57	-0.89	-0.86	-0.72	-0.79	0.43	0.64	0.84	1

Supplementary Figures

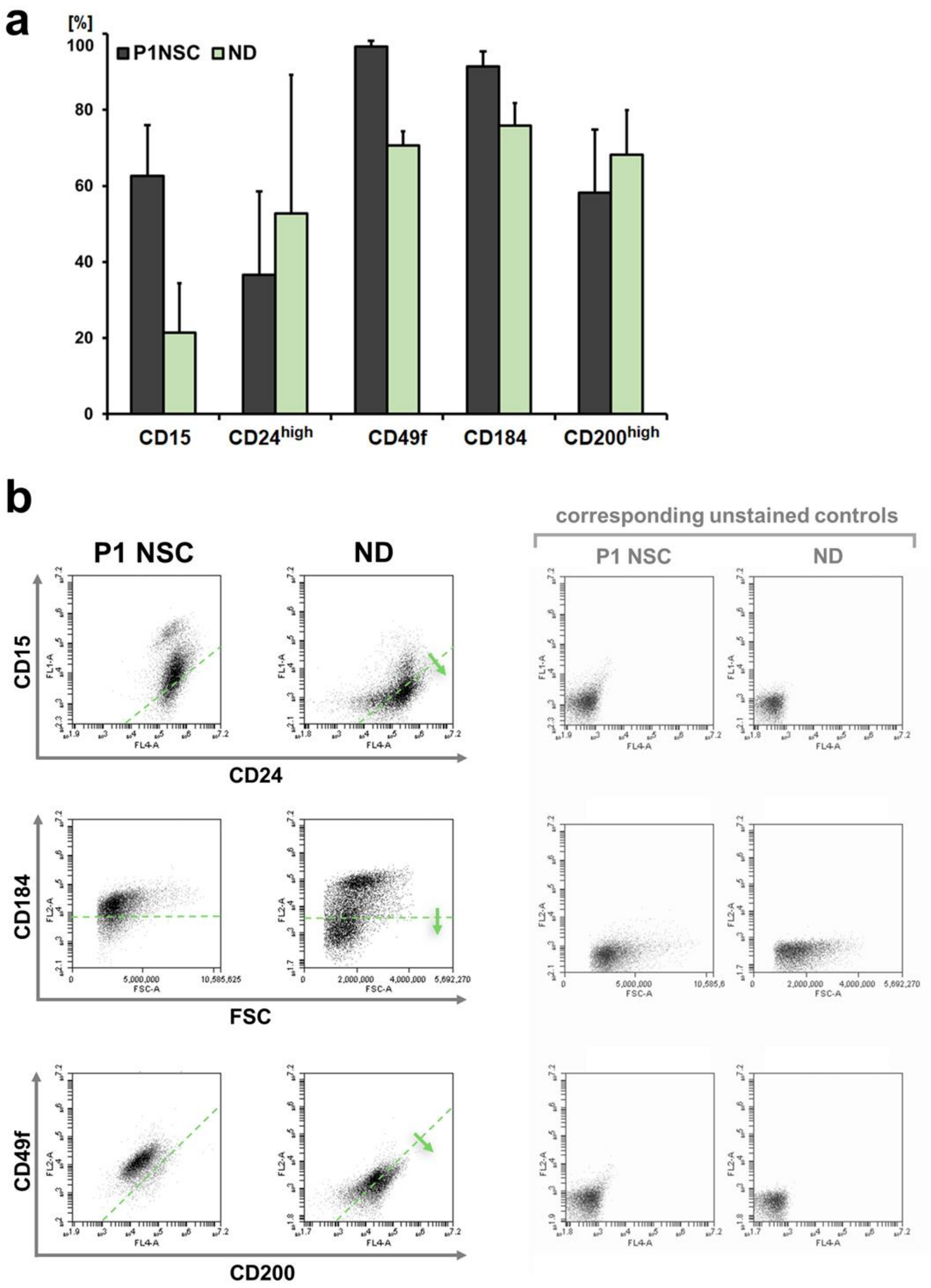

Suppl. Fig. 1: (**a**) Surface marker expression für CD15^{+}, CD24^{high}, CD49f^{+}, CD184^{+} and CD200^{high} at P1 neural stem cell (P1 NSC) versus neuronal differentiation (ND) stage are shown (n≥3 independent experimental repeats; error bars indicating standard deviation. (**b**) Bivariate flow cytometric dot plots comparing subpopulation shifts CD15/CD24, CD184, and CD49f/CD200 (representative experimental repeat of the above series; right panels: corresponding unstained controls as a reference point). Dotted lines added to facilitate visualization. Arrows indicate main directionality of subpopulation shifts upon neuronal differentiation: enhanced CD15^{-}/CD24^{high}, increased CD184^{-} subset, CD49f^{-}/CD200^{high}, confirming previous reports (see Pruszak *et al.*, 2009; Yuan *et al.*, 2011; Turaç *et al.*, 2013).

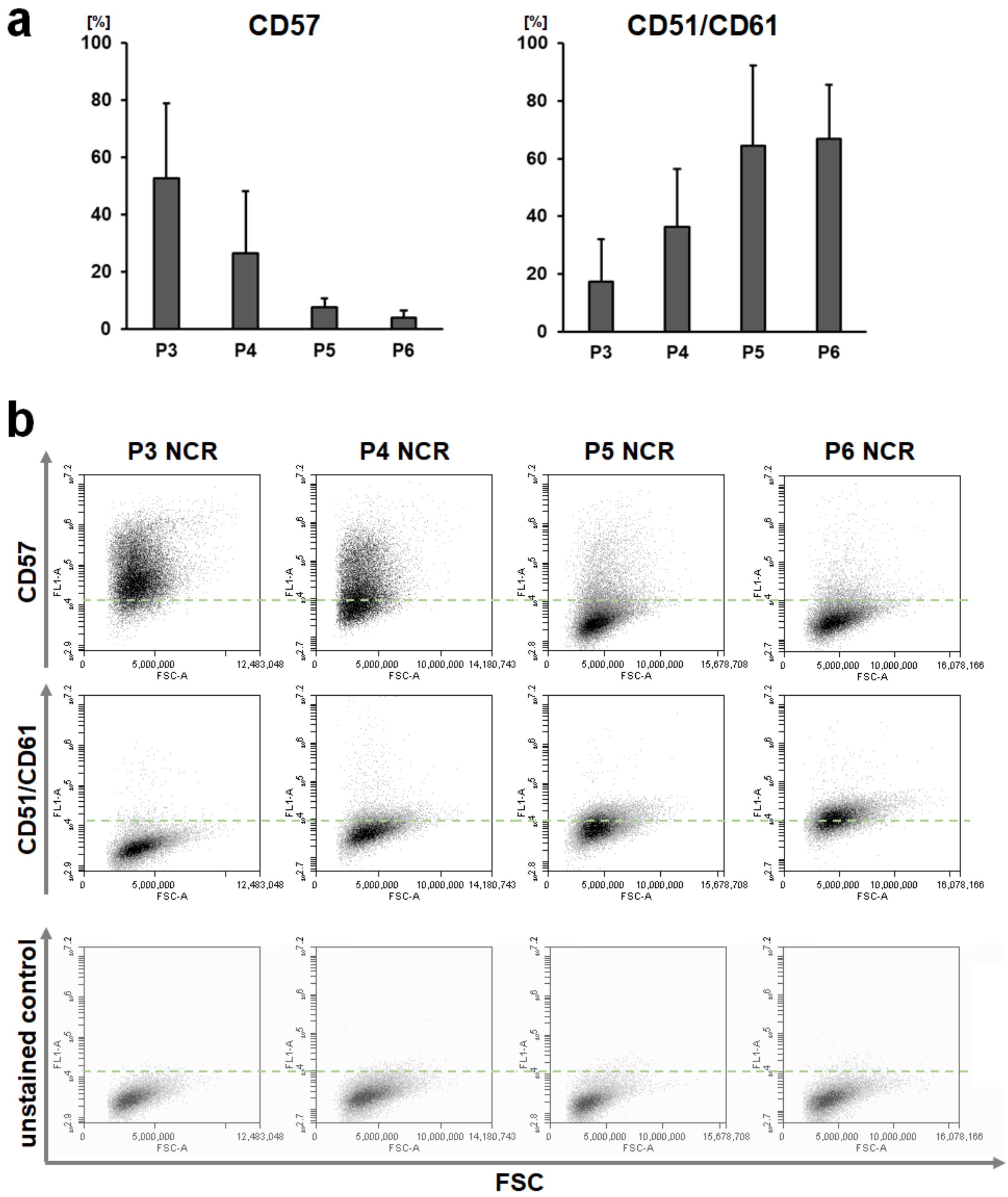

Suppl. Fig. 2: (**a**) Bar graphs depicting percentage of surface antigen expression for CD57 (left) and CD51/CD61 (right panel) positive fractions over the course of NCR passaging in vitro, P3 to P6, the period exhibiting the most profound phenotypic changes (n≥4 independent experimental repeats). (**b**) Flow cytometric dot plots showing overall decrease of the glycan epitope CD57 (top) and of the integrin CD51/CD61 (mid row) antigens over the course of P3 to P6. Bottom row shows corresponding unstained control samples acquired at the same voltage and shown with identical plot specifications. A representative experiment of the above series is shown.
